# Effects and mechanisms of Chinese herbal medicine for ulcerative colitis: Protocol for a systematic review and meta-analysis

**DOI:** 10.1097/MD.0000000000019768

**Published:** 2020-04-17

**Authors:** Qiaobo Ye, Zhipeng Hu, Maoyi Yang, Kaihua Qin, Yingguang Zhou

**Affiliations:** a Basic Medical College, Chengdu University of Traditional Chinese Medicine; b Hospital of Chengdu University of Traditional Chinese Medicine; c Health Preservation and Rehabilitation College, Chengdu University of Traditional Chinese Medicine, Chengdu, China.

**Keywords:** Chinese herbal medicine, meta-analysis, protocol, systematic review, ulcerative colitis

## Abstract

**Background:**

Ulcerative colitis (UC), an important type of inflammatory bowel disease, is mainly characterized by persistent and diffuse inflammatory response of colonic mucosa. Many studies have explored the effects and mechanisms of Chinese herbal medicine (CHM) in UC animal models. However, there is no systematic review and meta-analysis to evaluate and summarize these studies. The purpose of this study is to provide precise evidence of the effects and mechanisms of CHM in treating UC.

**Methods:**

Six databases, including 3 English databases and 3 Chinese databases will be searched. Two researchers will independently select eligible studies by reading titles, abstracts, and full texts according to the inclusion and exclusion criteria. Risk of bias assessment will be conducted by 2 independent reviewers using SYRCLE's risk of bias tool. The outcomes include total clinical effective rate, adverse events, disease activity index, interleukin-1β (IL-1β), IL-4, IL-6, IL-17; colombosa damage index, colonic mucosa damage index; myeloperoxidase; epidermal growth factor; transforming growth factor-β1; and histopathological score. Heterogeneity between studies will be assessed by Cochrane *X*^2^ and *I*^*2*^ tests. We will conduct subgroup analysis to explore the subgroup effects. We will also evaluate the stability of the results through sensitivity analysis and publication bias through funnel plot and Egger test.

**Results:**

The results will be published in peer-reviewed journals.

**Conclusion:**

This study can help us to understand the effects and possible mechanisms of CHM for UC. For further clinical researches, this study can help us to better look for possible effective medicines for clinical use.

**OSF registration number:**

DOI 10.17605/OSF.IO/YU5FN.

## 1 Introduction

Ulcerative colitis (UC) is a relapsing nonspecific inflammation condition that usually manifests as abdominal pain and diarrhea.^[1–3]^ With the improvement of living standard, the incidence of UC is increasing.^[4]^ Many complications occur in the later stage of UC, including toxic megacolon, intestinal perforation, lower gastrointestinal hemorrhage, intraepithelial neoplasia, and colorectal cancer.^[5,6]^ The recurrence of UC brings a heavy economic and health burden to the health system and patients.

At present, the management of UC includes 5-aminosalicylic acid, glucocorticoids, immunosuppressants, biological agents, and surgical treatment. In addition, fecal microbiota transplantation is considered to be a promising treatment for UC patients.^[7]^ However, most of these treatments have limitations in side effects and safety, such as drug dependence, adverse effects, decreased immune function, cancer risk, and so on.^[8–11]^ Thus, new treatment is urgently needed.

Chinese herbal medicine (CHM) has long been used in treating UC.^[12]^ In recent decades, many studies focusing on the effects and mechanisms of CHM for UC has been carried out.^[13]^ In animal experiments, many commonly used clinical herbal medicines or formulas, such as Sanhuang Shu’ai decoction, Gegen Qinlian decoction, and so on, have been found to play a good role in alleviating the condition of UC, and the mechanisms of which are related to the inhibition of NF-κB and other mechanisms.^[14–17]^ However, there is a lack of comprehensive summary and evaluation of these studies.

In this study, we will systematically evaluate the preclinical evidences and summarize mechanisms of CHM in treating UC. This study can help us to understand the effects and possible mechanisms of CHM for UC. For further clinical researches, this study can help us to better look for possible effective medicines for clinical use.

## 2 Methods and analysis

### 2.1 Study registration

This study has been registered on open science framework and the registration number is DOI 10.17605/OSF.IO/YU5FN (registration information is available at https://osf.io/yu5fn). This study protocol is reported according to the guidelines recommended in the preferred reporting items for systematic reviews and meta-analysis protocols checklist.^[18]^

### 2.2 Inclusion and exclusion criteria

#### 2.2.1 Study design

Only research articles will be included in our research. Conference, abstract, and review articles will not be included, for there is no detailed information for analysis in these articles.

#### 2.2.2 Participants

Studies with an established animal model of UC will be included in our research. In vitro studies and clinical trials will be excluded.

#### 2.2.3 Types of intervention

All type of CHM, whether single herbal medicine or formula, will be included. There will be no restriction about treatment course, frequency, and dosage.

#### 2.2.4 Types of controls

The control group can use purified water, saline, same solvent or no treatment.

#### 2.2.5 Outcomes

Main outcomes:

1. Disease activity index.
2. Serum inflammatory cytokines (interleukin [IL]-1β, IL-4, IL-6, IL-17).
3. Colombosa damage index.
4. Colonic mucosal damage index.
5. Myeloperoxidase.
6. Epidermal growth factor.
7. Transforming growth factor beta 1.
8. Histopathological score.

There will be no limit about the measurement of results.

### 2.3 Study search

Three English databases including PubMed, EMBASE and Cochrane Library and 3 Chinese databases including China National Knowledge Infrastructure, Wanfang Data knowledge service platform, and VIP information resource integration service platform will be searched from its inception to March 2020 without language limitation. In addition, we will search conference articles, published systematic reviews, abstract articles, and references to find out more ongoing and unpublished researches.

We will mainly use the following terms to search: (“Chinese herbal medicine” OR “Chinese Herbal Medicine” OR “Drugs, Chinese Herbal” OR “Chinese Herbal” OR “Chinese herbal” OR “Medicine, Chinese Traditional” OR “Traditional Chinese Medicine” OR “traditional Chinese medicine” OR “Traditional Chinese medicine decoction”) AND (“Ulcerative Colitis” OR “Colitis, Ulcerative” OR “Idiopathic Proctocolitis” OR “Colitis Gravis” OR “Inflammatory Bowel Disease, Ulcerative Colitis Type”). The work will be independently conducted by 2 authors (Qiaobo Ye and Maoyi Yang). The search process of the PubMed is presented in Table 1.

**Table 1:**
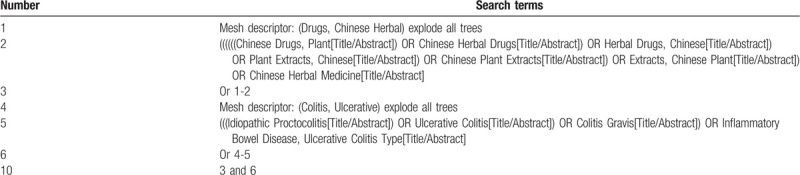
Example of PubMed search strategy.

### 2.4 Study selection

**Figure 1:**
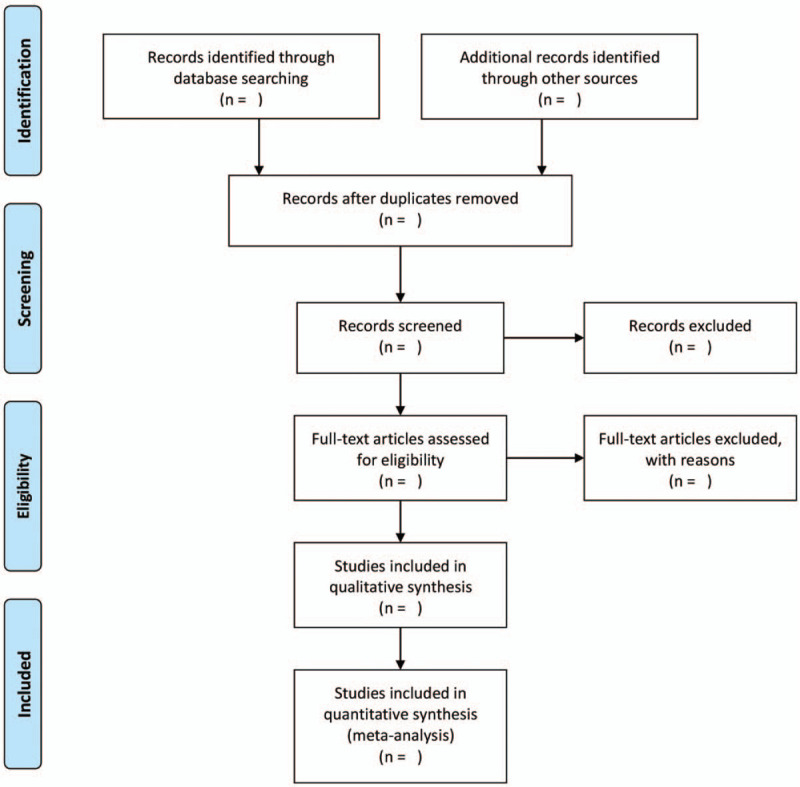
Flow chart of study selection.

We will use EndNote X8 for Mac to manage the citations. Two researchers (Kaihua Qin and Yingguang Zhou) will screen citations independently. Disagreement between 2 authors will be solved by discussion with a third author (Zhipeng Hu). A research flow chart will be drawn to show the whole process of research selection (Fig. 1).

### 2.5 Data extraction

The following data will be extracted: the first author's name, publication time, article title, species of experimental animals, sex of experimental animals, number of experimental animals, methods of experimental modeling, intervention measures of experimental group, intervention measures of control group, intervention time, efficacy data, and mechanism. For some outcomes, if the author only provide figure, then we will request the data from authors or extract the data from the figure using software.

### 2.6 Risk of bias assessment

The risk of bias will be assessed by SYRCLE's risk of bias tool. This tool is widely used in the risk of bias assessment for animal studies. In this tool, there are 10 domains:

(1) Sequence generation;
(2) Baseline characteristics;
(3) Allocation concealment;
(4) Random housing;
(5) Blinding (performance bias);
(6) Random outcome assessment;
(7) Blinding (detection bias);
(8) Incomplete outcome data;
(9) Selective outcome reporting;
(10) Other sources of bias.

Two authors (Qiaobo Ye and Kaihua Qin) will conduct the risk of bias independently. We will contact the authors for more detailed information if necessary.

### 2.7 Data analysis

Data analysis will be conducted in Review Manager Version 5.3 and Stata 14.0 software. Since all outcomes are continuous variables, 95% confidence interval and mean difference or standardized mean difference will be used to calculate the effect size. Cochrane *X*^2^ and *I*^*2*^ tests^[19]^ will be conducted to test the heterogeneity analysis between studies. When *P* > .05 and *I*^*2*^ < 50%, the heterogeneity can be ignored, and a fixed effect model will be used. When *P* < .05 and *I*^*2*^ > 50%, the heterogeneity is substantial and a random effect model will be used. If quantitative synthesis is not appropriate, then we will present the results in tables and figures. Finally, we will summarize the mechanisms of action of CHM in treating UC.

### 2.8 Investigation of heterogeneity

If there are adequate studies, then we will conduct subgroup analysis to explore the subgroup effects and source of heterogeneity.^[20]^ Subgroup analysis will be performed according to these factors: course of treatment (<1 week or >1 week), model of UC (2,4,6-trinitrobenzene Sulfonic Acid, dextran sulfate sodium, or others) and species (rats or mice).^[21,22]^

### 2.9 Sensitivity analysis

We will conduct sensitivity analysis to test whether the results are robust. We will conduct sensitivity analysis by excluding each research one by one. In addition, we also evaluated the impact of different analytical methods and effect models on the results.

### 2.10 Publication bias assessment

We will conduct funnel plot and Egger test to assess publication bias. If the funnel plot is asymmetric or *P* < .05 in Egger test, then there is publication bias.^[23]^

### 2.11 Ethics and dissemination

Meta-analysis is an analysis of previous research data and does not require ethical approval. The results of this study will be published in peer-reviewed journals.

## 3 Discussion

In recent years, due to the improvement of living standards and the change of lifestyle, the incidence of UC has increased dramatically. As the etiology and pathogenesis of the disease have not been fully elucidated, there is a lack of effective treatment. UC has been listed as one of the most refractory diseases by the World Health Organization.^[24]^ The main characteristics of CHM are multi-component, multi-target and synergism, which is quite different from western medicine. In China, CHM has been used to treat UC for thousands of years. Many researchers are trying to find out the best medicine or prescription for UC and its related mechanisms. However, there is no meta-analysis to systematically evaluate these research evidence and summarize it mechanisms.

This study will systematically summarize the research evidence and mechanisms of CHM in treating UC, which will be of great help to further drug research and clinical trials in the future. In this study, we will take the following measures to ensure the reliability of the research results.

First of all, we will collect relevant research results extensively, which will ensure that our research results are as comprehensive as possible. Second, this work will be carried out independently by 2 researchers, which can ensure the transparency of the research process. In terms of data analysis, we will conduct a comprehensive analysis of the data, including a detailed sensitivity analysis, which can ensure the credibility of the result course.

## Author contributions

**Conceptualization:** Qiaobo Ye, Maoyi Yang, Zhipeng Hu.

**Data curation:** Qiaobo Ye, Maoyi Yang.

**Formal analysis:** Maoyi Yang, Zhipeng Hu.

**Investigation:** Qiaobo Ye, Kaihua Qin, Yingguang Zhou.

**Methodology:** Qiaobo Ye, Maoyi Yang, Zhipeng Hu.

**Project administration:** Zhipeng Hu.

**Software:** Qiaobo Ye, Kaihua Qin.

**Visualization:** Kaihua Qin.

**Writing – original draft:** Maoyi Yang.

**Writing – review & editing:** Qiaobo Ye and Yingguang Zhou.
